# Supplementary material for: The Construct CoSe2 on Carbon Nanosheets as High Sensitivity Catalysts for Electro-Catalytic Oxidation of Glucose
Source: Nanomaterials (Basel). 2022 Feb 7;12(3):572. doi: 10.3390/nano12030572 (PMC8838914; doi:10.3390/nano12030572)
Supplement: Supplementary file 1 [file nanomaterials-12-00572-s001.zip › nanomaterials-1563406-supplementary.pdf]

Supplementary Materials

The Construct $CoSe_2$ on Carbon Nanosheets as High Sensitivity Catalysts for Electro-Catalytic Oxidation of Glucose

Di Wang [1, 2] and Ying Chang [1,*]

[1] Inner Mongolia Key Laboratory of Green Catalysis and Inner Mongolia Collaborative Innovation Center for Water Environment Safety, College of Chemistry and Environmental Science, Inner Mongolia Normal University, Hohhot 010022, China; 2120200811@mail.nankai.edu.cn

[2] Key Laboratory of Advanced Energy Materials Chemistry (Ministry of Education), Renewable Energy
Conversion and Storage Center (RECAST), College of Chemistry, Nankai University, Tianjin 300071, China

* Correspondence: changying@imnu.edu.cn

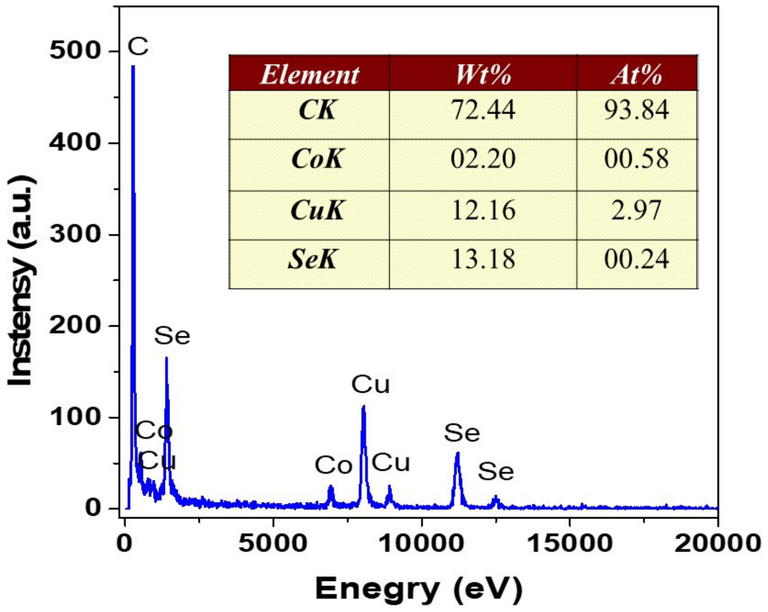

Figure S1. EDS patterns of $CoSe_2$-400 nanomaterial.

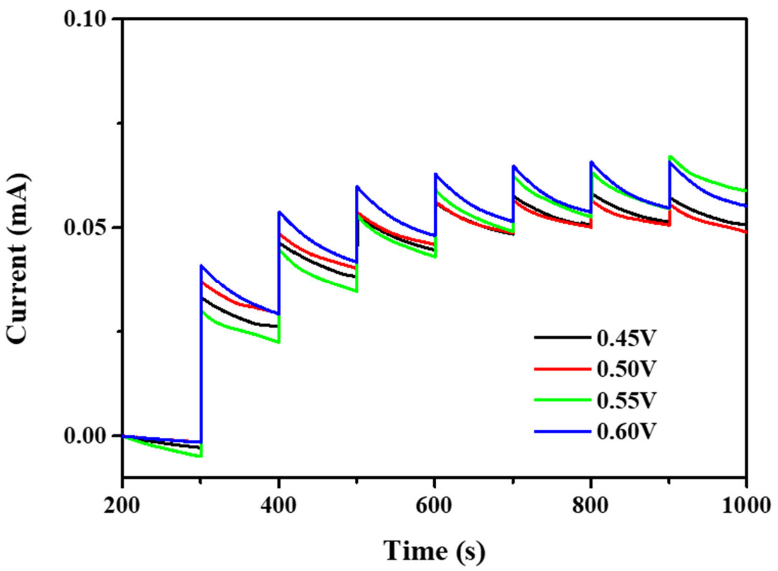

Figure S2. I-t curves at different applied voltages of $CoSe_2$-400 nanocomposite, the current at 200s is as the backgroud.
